# Generation Z, values, and media: from influencers to BeReal, between visibility and authenticity

**DOI:** 10.3389/fsoc.2023.1304093

**Published:** 2024-01-10

**Authors:** Simona Tirocchi

**Affiliations:** University of Turin, Turin, Italy

**Keywords:** Generation Z, values, BeReal, influencers, digital literacy

## Abstract

This study examines the connection between values perceived as important by Generation Z and the values conveyed by the media contents chosen and consumed by young individuals. The article’s main objective is to identify the values that a sample of Italian university students, aged between 20 and 23, consider most significant. It explores their perceptions and expectations regarding contemporary society and the ethical trends therein. Furthermore, it investigates their preferred media and the values they believe these media platforms convey, trying to investigate the relationship between values and favorite media. Media and digital platforms, indeed, play an increasingly vital role in shaping and disseminating values. YouTubers, influencers, or, in a broader context, content on social media platforms (such as Instagram, TikTok, as well as entertainment platforms like Netflix) demonstrate how the new mediators of communication and the forms of media content favored by young individuals are, more evidently than ever, intertwined with the sharing of values, norms, and social expectations, much like family and school once were. One example that emerged from the research concerned the success of the “Bereal” platform, linked on the one hand to the desire to make public and share one’s self-image, and on the other hand to the need to show oneself as “authentic.” Through the conduct of five focus groups in December 2022 involving 60 university students from the University of Turin, this research reveals a substantial continuity in the values considered most important by young individuals (compared to previous national surveys). The article also demonstrates how the values conveyed by the media favored by young people do not always correspond to traditional ones and express needs that, at times, the new digital platforms and their protagonists manage to intercept.

## Introduction

The study discussed in this article focuses on a classic theme of sociological analysis, that of values, which takes on renewed significance in our increasingly complex yet fragile societies. These societies are characterized by a proliferation of expressive forms, the effects of globalization, and ethical relativism. However, the approach taken to examine values in this study departs from traditional methods. It aims to establish a connection between the values held by young individuals, particularly a sample of Generation Z, and those propagated by their preferred media outlets, encompassing both traditional media and the novel communicative forms produced by digital platforms.

Following a concise review of the relevant literature concerning the concept of value, youth values in the Italian context, and the increasingly close and immersive relationship between young people and digital platforms, this article presents a qualitative research study conducted through the focus group technique. The research has a dual purpose: first, to provide an overview of the values and preferred media content of young individuals, along with a portrayal of the society in which they reside, reflecting these very values. Second, to establish a connection between the value system of young people and the values expressed and propagated by influencers, digital creators, web pages, and TV series.

The anticipated conclusions prompt reflection on the necessity of sensitizing young individuals (as well as adults) to conscious media consumption, particularly in an era characterized by the emergence of applications such as “Bereal,” which emphasizes the importance of “authenticity,” as well as the compulsion to document every moment of one’s daily life.

### Theoretical framework—values and youth citizenship

An increasingly relevant aspect for the sociological study of younger generations pertains to the values they share and consider most important. This analysis should be of considerable significance as it underscores the intrinsic relationship between the values held by young individuals and citizenship. Values are indeed related to the education and the acquisition of citizenship by the younger generations (Tonge et al., 2012; Brown and Smith, 2015).

Indeed, these values serve as a reflection of their worldview, priorities, and aspirations within the society in which they reside. Moreover, values play a pivotal role in shaping their actions and exert a profound influence on the trajectory society will follow.

In the long tradition of research on the ethical dimension, various authors have made significant contributions. It is crucial to distinguish between values and attitudes: values are distinct from attitudes in that a value is a context-independent proscriptive or prescriptive belief, whereas attitudes are descriptive, prescriptive, or evaluative beliefs that focus on a specific object (Rokeach, 1973; Braithwaite and Scott, 1991). According to Rokeach (1973), a value is an “enduring belief that a specific mode of conduct or end-state of existence is personally or socially preferable to an opposite or converse mode of conduct or end-state of existence” (p. 5). In his analysis, Rokeach also considers the value system, which is “an enduring organization of beliefs concerning preferable modes of conduct or end-states of existence along a continuum of importance” (p. 5).

Another values-based approach is the theory of Schwartz (2006), which regards values as emotionally charged beliefs expressing specific or general interests developed in all human societies to satisfy three fundamental needs of individuals and communities: individual biological needs, the necessity of establishing coordinated social interactions, and the needs for survival and well-being of collectives (Schwartz and Bilsky, 1990). Values refer to desirable goals, serving as criteria that guide the selection or evaluation of actions, policies, individuals, and events, motivating action based on their relative importance attributed by individuals and/or societies, thus leading to the development of distinct hierarchical systems of value priorities. Schwartz’s theory postulates the existence of an integrated system of 10 universal value types, characterized by the same meaning across all cultures: Power, Achievement, Stimulation, Hedonism, Self-Direction, Universalism, Benevolence, Tradition, Conformity, and Security.

Today, however, the issue of values assumes renewed importance in a society where the certainties of modernity appear to have crumbled, giving way to situations of great fragility and existential uncertainty (Giddens, 1991; Bauman, 2000, 2007; Stehr, 2001; Rosa, 2010). The processes of globalization, which had already profoundly altered the fabric of society by accentuating cultural differences while simultaneously leveling them, have been overtaken by the impact of the pandemic, which has disrupted all previous structures, both at the micro and macro levels. The COVID-19 pandemic, indeed, has had an impact on numerous dimensions of daily life, particularly for young people. Changes have occurred, especially in the relationship between public and private spaces and in the dimension of social relationships. In these areas, digital platforms have played a significant role, providing a space for reshaping one’s self (Tirocchi et al., 2023) and engaging in various forms of subjectivity (Tirocchi and Serpieri, 2020).

Reflection on the values of young people must also consider the fact that socialization has become, since modernity (and even more so in postmodernity), a dynamic, open, and unstable process that lacks a fixed endpoint and does not conclude at a specific moment (Garelli et al., 2006; Buckingham, 2007). Furthermore, it no longer coincides with the mere internalization of norms and values but is configured as a negotiation process in which the individual exercises agency. In the face of these changing processes and in a context of social pluralism and complexity, there is also a pluralization of value references, which are increasingly less influenced by institutionalized practices and more by informal and intersubjective dimensions based on peer-to-peer sharing, emphasizing the horizontality of social relationships and the entry of new agencies into socialization processes, such as the media.

Within the tradition of research on values, the contribution of Ronald Inglehart is noteworthy, who, through longitudinal research, documented the transition in post-industrial societies from materialist values to post-materialist values. This gradual change, labeled the “silent revolution,” is also linked to achieving economic and material security, enabling (for younger cohorts) a greater emphasis on values related to free self-expression, the promotion of quality of life, esthetic satisfaction, and central aspects such as the sense of community or the environment (Inglehart, 1977, 1997, 2000).

Regarding other considerations on studies and research on the values of young people in the Italian context, it can be observed that the narratives they propose are frequently misleading and do not accurately reflect their “real” world. As highlighted by Alessandro Cavalli (2007), generations often become the object of projections from the adult world, which appears to unload its deficiencies onto them. These projections primarily affect the areas of values, norms, and future orientation, which, in the eyes of adults, appear increasingly uncertain. In this regard, young people are depicted within a framework of economic and existential fragility that sets them apart from previous generations. In the cognitive horizon of young people, a vision of the future emerges as indefinite and unpredictable: alternatively, open to any event or none at all (Leonini and Spanò, 2015). Adolescents and young people display complex and risky trajectories of self-construction deeply influenced by extended transitions to adulthood, characterized primarily by uncertainty and a valorization of restricted sociality less open to the broader social context. This has revealed both the difficulty in building a future orientation and external engagement and, at the same time, a proactive tension toward the future, oriented toward learning to exist and be in the world, defining original strategies for self-appropriation and biographical construction (Besozzi, 2009; Merico, 2009a).

At this point, it is appropriate to offer a review of the scientific literature on values considered important by young people, analyzing trends and changes observed over the years. From the tradition of analyzing the values of Italian youth, particularly pursued by the IARD Institute since the 1980s, in the period from 1983 to 2004,^1^ among young people aged 15 to 24, “family stability” emerged as the most important value (De Lillo, 2007). “Work” occupied the second position but showed a decrease in significance, as did “friendship,” although the entire sphere of “close social relations” gained ground.

In 2007 (based on a survey conducted on values declared in 2004), “health” prevailed in the top position (introduced for the first time in that survey), followed by “family” and “peace.” In the study conducted by Garelli et al. (2006), “family” was chosen as the first value by 57% of respondents, while “friendship” ranked second with 29.7%. In the aforementioned research on the transition to adulthood among young people, “being successful” was identified as an important value by one-third of the interviewees, while in terms of the transition to adulthood, “responsibility” (toward oneself and others) and “autonomy” were considered particularly important (Besozzi, 2009; Merico, 2009a,b).

The results of the latest surveys of young people conducted in Italy, especially by the Toniolo (2021, 2022),^2^ have inevitably been influenced by the impact of the COVID-19 pandemic, an event that profoundly affected societies worldwide, catching them unprepared, substantially altering social habits, and requiring a reorganization of resources and interpersonal relationships. From a data perspective, it can be observed that the percentage of those claiming to have a “positive self-idea,” in the categories “very” or “extremely,” decreased from 53.3% in 2020 to 45.9% in 2022. Similarly, “motivation and enthusiasm in one’s actions” decreased during the same period, from 64.5% to 57.4%, while the perception of “pursuing a goal” declined from 67% to 60%. The conflict in Ukraine represented another event that significantly impacted young people’s lives, increasing perceived levels of risk. Approximately 27% of Italians, Spaniards, and Britons and 38% of Germans, and 21% of the French, indicated a high perceived risk (scores of 4 or 5 on a scale from 0 to 5).^3^

Among the latest trends evident in this recent study by Toniolo (2023), alongside uncertainty about the future, there is a hope for “improvement” and “openness to change.” There is a high demand for “a job with an adequate income” (68% of young people aged 18 to 22), but there is also a desire “to work within a company that shares their values” (60%) and “engages in activities with positive impacts on society and the environment” (60%). Awareness of the National Recovery and Resilience Plan (PNRR^4^) is very low: 31.8% do not know what it is, but among those who are informed, 59.9% of young people aged 18 to 22 agree “somewhat” or “very much” that it can help solve the country’s structural problems and contribute to a revival of growth, as well as improve opportunities for young people (52.2%).

### Generation Z, media, and values

Another fundamental aspect characterizing young people globally is their relationship with the media. The media, in turn, convey imaginaries and values and exert a significant impact on the younger generations, as they are constantly exposed to messages, images, and narratives presented through various media outlets and channels. The way in which the media present ideas and values naturally influences how they perceive the world, as the media have the power to construct social reality, as demonstrated by numerous theories.^5^ Social discourses on the relationship between media, values, and new generations (often based on commonplaces rather than scientific research) have always attempted to demonstrate that television programs, films, and video games can promote values such as individualism, aggression, performance anxiety, premature sexualization, and substance abuse. However, this perspective seems to overlook the complexity of interconnections and relationships.

When we speak of young people, we are specifically referring to the so-called “Generation Z.” With this defining label, we refer to a generation that came after the Millennials and includes individuals born from the late 1990s to the mid-2010s. However, as also stated elsewhere (Dimock, 2019; Aroldi and Colombo, 2020; Tirocchi et al., 2022),^6^ this is a definition that should not be used deterministically, thinking that it has fixed and non-negotiable characteristics.^7^

Numerous assumptions have been made regarding the emerging Generation Z, giving rise to the development of various enduring “myths” (Bogacheva and Sivak, 2019). These myths are linked to the challenges of studying this generation during its formative years, which coincided with a profound societal transformation as we transitioned to a new phase of civilization (Bresler, 2023). Research reveals distinct cultural traits of this generation, such as their interconnectedness within a network of solidarity, encompassing not only their immediate family but also extended relatives and friends with whom they maintain constant interaction. The concept of family takes on a broader meaning, fostering interpersonal connections that influence their interactions beyond the community. In Generation Z, the nuclear family evolves into a closely-knit community, characterized by strong familial, friendship, and professional bonds. Within this community, Generation Z predominantly seeks common values, consciously avoiding contentious topics (Bresler, 2023).

Generation Z is often referred to as “the generation of metamodernism,” according to Van den Akker et al. (2017). Members of this generation perceive metamodernism as a cultural phenomenon that arises from the rapid societal changes, leading to the transformation of institutions and organizations. Previous stages of civilization’s values persist but adapt to the demands of modern times.

Additionally, Generation Z views historical and cultural heritage and contemporary culture as a non-deterministic cultural landscape—a simulacrum of universal culture shaped by the values of both the emerging digital era and earlier stages of civilization development (Bresler, 2023). Bresler’s research on Generation Z’s perception of cultural heritage demonstrates a positive attitude toward various styles in both classical and modern music and visual arts. In contrast to conventional beliefs, this generation does not filter various art genres through the lens of youth subculture determinism, but instead, they see it as a broader cultural information repository. The typical divisions between supporters of different musical and visual genres and styles of fine art are not prevalent among a significant portion of Generation Z. This characteristic can be attributed to their immersion in the digital environment and the unique characteristics of the present information-based society.

Finally, Yunissov et al.’s (2023) research aims to ascertain the perspectives of Kazakh Generation Z students on achieving success in modern life and their connection with moral values. The authors conclude that young Generation Z individuals in Kazakhstan still hold onto traditional cultural values and consider them essential for attaining success. However, these traditional values are not fixed and may evolve depending on the professional and regional socialization environment experienced by the youth of Generation Z.

It is, however, a generation that has made media its distinctive feature because it was born at a time when they became an indispensable reality and an important environment for the construction of identities, almost on par with face-to-face interaction. In this regard, scholars have spoken of the “onlife” dimension (Floridi, 2014) and the coalescence between online and offline dimensions (Boccia Artieri et al., 2017), highlighting the inseparable interweaving of the digital dimension and what we still call the “real” world.

Furthermore, Generation Z emerges on the social scene at a time when the media landscape has been characterized by the revolution of social media, a historical phase in which we transitioned from a static web to a participatory web (web 2.0). This shift increasingly placed the consumer’s ability to generate their own content at the center (the so-called “prosumer” and then “produser”^8^). It is also part of the new infrastructure of the platform society (Van Dijck et al., 2018), where a significant portion of people’s daily lives is governed by platforms. Digital platforms have become spaces of expression, not only for the collective production of meaning, identity construction, engagement, and political participation but also for accessing a range of opportunities (boyd, 2014; Stahl and Literat, 2022). They also serve as a means of informal skill acquisition (Scolari, 2018; Tirocchi and Taddeo, 2019; Tirocchi and Serpieri, 2020; Taddeo and Tirocchi, 2021) and participation in new opportunities beyond the limitations of physical spaces, as demonstrated by the period of the pandemic (Tirocchi et al., 2023). This is not to deny that platforms can also be the site of negative experiences such as cyberbullying, stress, apathy, not to mention all the issues related to privacy and big data management (Gangneux, 2019; Fu and Cook, 2020).

As indicated by research from the Pew Research Center (2022)^9^ in the U.S. context, TikTok has risen since 2014–2015, while Facebook usage has declined, and Instagram and Snapchat have grown among young adolescents. Similarly, an analysis conducted as part of a study on the perception of cyberviolence by Generation Z in Italy reveals that the most commonly used platforms among young people are WhatsApp and Instagram, with “every day” usage being prevalent at 98.9% for the former and 92.1% for the latter (Tirocchi et al., 2022).

Research published by the We Are Social, Hootsuite (2022), presents data in agreement with those just illustrated: out of approximately 43 million social media users in Italy, the top three platforms used monthly were WhatsApp (90.8%), Facebook (78.6%), and Instagram (71.4%), while the top two preferred platforms were WhatsApp (39.7%) and Instagram (21.7%).

With these scenario premises, the data analyzed in the following pages will illustrate the direction in which the media preferences of young people are heading and the values they convey.

## Materials and methods: conducting focus groups

The two main research questions investigated in this article are as follows:

*RQ1: Which values are considered most important by Generation Z (especially young adults)?*

*RQ2: What are their favorite media and what values do they convey?*

The aim is to analyze the proximity or divergence between these two dimensions: personal values and those conveyed by the media platforms they most frequently use and engage with.

To investigate these aspects, a qualitative methodology was employed. Specifically, in December 2022, five focus groups were conducted, involving a total of 60 male and female students enrolled in the Bachelor’s Degree in Education Sciences at the University of Turin.

The sample consisted of 57 females and 3 males aged between 20 and 23 years. The focus groups, homogeneous in their composition, had a duration of approximately 1 h each and were conducted in the premises of the University of Turin. They were moderated by a senior researcher (with over 20 years of experience in social research) and expert in the subject matter of the investigation.

Based on the research objectives, the purpose of these focus groups was to bring to light, on one hand, the values of the young people, and on the other hand, the media preferred by the young people and the values conveyed by them.

The choice of using focus groups as a research technique was informed by its well-established and tested utility in the field (Morgan, 1998; Acocella, 2012), including within media studies (Lunt and Livingstone, 1996). Consequently, this study served as an opportunity to employ the focus group method to analyze the nexus between media and values, given its effectiveness in collecting data on the perceptions and attitudes of young individuals. Indeed, focus groups provide a wealth of data stemming from participant interactions and the emergence of diverse opinions.

Participants were selected through a convenience sampling approach (Krueger and Casey, 2000; Stewart and Shamdasani, 2014), with criteria set for inclusion in the study. The primary criterion was age (individuals belonging to the Generation Z cohort), while being media consumers was not a mandatory requirement.

It should also be noted that the sample in question cannot be considered representative of the Italian population, as it refers to a local context (the University of Turin, located in the Piedmont Region), also considering the socio-cultural characteristics of the sample.

Lastly, all subjects participated voluntarily.

The focus group guideline encompassed the following thematic areas, partially coinciding with the research questions:

*Contemporary Society—How do young people define the current society?*
*Values—What are the values they consider most important for themselves and their lives?*
*Media—What are the preferred and most-used media platforms among young individuals, and which values do they believe are predominantly conveyed by media content? What media content (or characteristics of content) do they wish to see more of or find lacking within the available media contents?*

In each focus group, 12 participants were involved. The sessions took place on the university premises, with participants seated in a circular arrangement around a long table (circle seating). Each participant had a name tag placed in front of them to facilitate the transcription of their contributions.

The moderator aimed to establish a permissive, warm, and friendly environment while exercising an unobtrusive control. Name tags were provided to promote the recognition of individual contributions for the final transcription.

An introduction was given by the moderator, clarifying the focus group’s objectives and the intended use of research data. Participants were informed that there were no right or wrong answers, only differing points of view. Throughout the focus group discussions, the moderator made efforts to engage even the more reserved participants, using an engaging communication style and providing examples to clarify questions. There was, in general, a high level of interest from the participants in the subject matter. Some participated more significantly, offering many insights to the discussion, while others were shyer and were invited by the moderator to express their opinions.

On two occasions, participants were asked to create lists on white cards provided by the moderator. In the first instance, they were instructed to create an ordered list of the five values they considered most important, and in the second, they were asked to identify their three favorite media contents (e.g., personalities, influencers, websites, social media pages, songs, movies, TV series, etc.).

Students encountered some difficulty in understanding the concept of “values,” but were subsequently informed that these were labels, nouns (e.g., love, friendship, etc.), and not examples or explanations of values. To help identify values within the media, examples related to these aspects were provided (e.g., “What values does the reality show ‘Big Brother’ convey?” Or “What values does a specific Instagram page convey?”).

The focus group sessions were audio-recorded and later transcribed meticulously, considering both verbal and non-verbal aspects. Notes taken during the sessions supplemented the recordings.

Data analysis was carried out using thematic analysis (Boyatzis, 1998; Braun and Clarke, 2006), aided by a grid that facilitated the synthesis of diverse perspectives within each thematic area. Comparisons and potential contrasting results among individual focus groups were considered during the analysis.

Qualitative depth was enhanced by incorporating quotations and comments from the discussions, providing a better contextualization of participants’ choices. Additionally, the final analysis considered the preferences expressed by participants on the cards, as mentioned earlier, which complemented the group discussions.

## Results

### The society of superficiality: perceptions of contemporary social reality in Generation Z

In delineating contemporary society, participants in the focus groups articulate a *dynamic* [M (f)], *fast-paced*, and *rapidly changing* [A (f)] environment where the constant pursuit of being “first” prevails [C (f)]. They have grown accustomed to instant gratification, feeling the need to keep up with the times, as falling behind, for instance in fashion trends, is seen as a personal descent [A (f)]. It’s a society that prioritizes the fleeting over the enduring. As one participant noted, “My grandparents bought only two outfits and stayed together for life, but now it seems like change happens faster, and we feel like we never have enough time” [G (f)]. Another participant added, “I think about lunch; it seems like people do not have time to eat or prepare anymore” [D (f)].

Participants also describe this society as *multicultural* [L (f)], *complex*, yet rife with inequities and contradictions. Some see it as highly *individualistic*, with “a lack of a sense of community” [G (f)], where the focus is overwhelmingly on the individual. The pandemic exacerbated this sense of individualism, as “university was experienced individually, ‘I study’, but now it seems like a community has [re]formed.” They also discuss a *materialistic* society, where personal gain, money, career, and self-presentation take precedence, often leading to selfishness [C (f)].

Moreover, with reference to the previously mentioned characteristics, a common thread emerges: the perception of a *superficial* society that has lost depth and meaning. “There is so much superficiality; we cannot grasp the depths of other people. There is so much emphasis on appearances, from friendships to the workplace. In a group of friends, we attach ourselves to those liked by all, following those who are more influential” [G (f)]. This aspect is linked by the youth to the influence exerted by social media, which increasingly emphasizes image, creating a perilous overlap between a person’s identity and their representation. Furthermore, “we only want to see the beautiful side of people, without thinking that there might be sad moments” [C (f)]. For this society, it’s essential to focus only “on self-realization and pay little attention to what’s around” [L (f)]. As another participant asserts, “In this society, the problem is that we no longer delve deep into people; we are superficial… We immediately erase anything we do wrong… In this sense, it could also be called a ‘society of forgetfulness’” [L (f)].

While self-promotion aligns with showcasing physical appearances on social media, the youth are aware that media representations are often nothing more than a staged portrayal of a more attractive and desirable life than reality. They frequently post daily activities on social media just to be observed and “envied” by their peers.

One interviewee reflects, “in high school, I used to help a disabled boy and would go get lunch with him. I was perceived as the one who helped the ‘outsider’. At some point, I started wondering if this gesture was right, if I could stop doing it because my classmates might judge me negatively” [C (f)].

According to other interviewees, it’s a society of prejudice where people often stop at their preconceived notions about others [M (f)]. It’s a society where “anything less than perfect is immediately judged” [S (f)].

In their discussions, the youth also describe an environment where relationships seem to be perceived as “chains,” highlighting a clear contrast between a society of control (Foucault, 1979) and individual freedom, the so-called “agency” (Giddens, 1991). Thus, the image of a risky society reemerges, where the individual bears the full weight of constructing their individual identity (Beck, 1992), and the proliferation of opportunities can complicate matters further.

Another aspect emphasized in this society concerns gender issues, with young adults perceiving that gender equality has not been effectively achieved, at least in Italy. They see it as a patriarchal society [F (f)], where “men earn higher salaries than women” [A (f)], and where it’s challenging to balance a career with having children.

In the perception of the youth, LGBT issues are not discussed enough: “In Italy, we are still behind; our mindset is too old-fashioned because there are elderly people in government, so politicians do not talk about these things” [F (f)]. “Young people are more open to the LGBT community, which the elderly view unfavorably… Social media, even Instagram, interacts more with our generation; we are more inclusive… Someone from the 1960s would not openly declare their homosexuality” [A (f)]. The youth feel the need for controversial topics, such as homosexuality or abortion, to be addressed by the media.

For these reasons, the interviewees describe this society as static: “Even though there are pushes for change, we always end up back where we started. For example, with the euthanasia referendum… there’s a push for innovation, but then we regress” [F (f)].

Some participants also feel the need to distance themselves from the false and glossy world of influencers prevalent on social media. According to one of them, a true influencer is not just “someone on social media.” Influential, in this sense, can also be a friend: “For me, they are very influential because they help me grow. They are important because we talk about everything. We go for walks on the bike path, or we meet up at catechism; three of my closest friends teach catechism with me” [S (f)].

Compared to the past, this society seems to lack reference points. “Even with friends, with people close to us, there is no value for respect or courtesy. Parents no longer exercise a controlling function” [F (f)]. “School and family have changed. In the past, there was the teacher, and people always used formal language. Today, we see more news about students overturning desks or threatening teachers with knives to pass. Families used to be more united, at least from what we hear; there was mutual help among families. Now, the family unit is less cohesive, and there is less support than there used to be” [E (f)]. “Previous generations (e.g., those of ‘68’) took to the streets, believed in values, and sought change; now, these values are absent in society. It’s a constantly changing society… Due to individualization, we no longer feel these values, we no longer feel like a group” [E (f)].

The youth also reflect on the impact of the pandemic, which, instead of improving, has worsened societal attitudes, not only toward helping others but also in interpersonal relations. As one participant noted, “Instead of improving us, the pandemic has made us worse, both in terms of helping others and in how we interact with people. Volunteering, I notice that people are more hesitant now; they used to be more open” [L (f)].

### The most important values in the lives of Generation Z

During the focus group sessions, participants were asked to identify the most important values in their lives and list the top 5 in order of importance on a designated card provided at the beginning of the session. One interesting initial observation was that adolescents had some difficulty even in pinpointing their most important values, as if they were struggling to conceptualize what still constitutes a value in today’s society, marked by uncertainty and ethical relativism.

**Figure 1:**
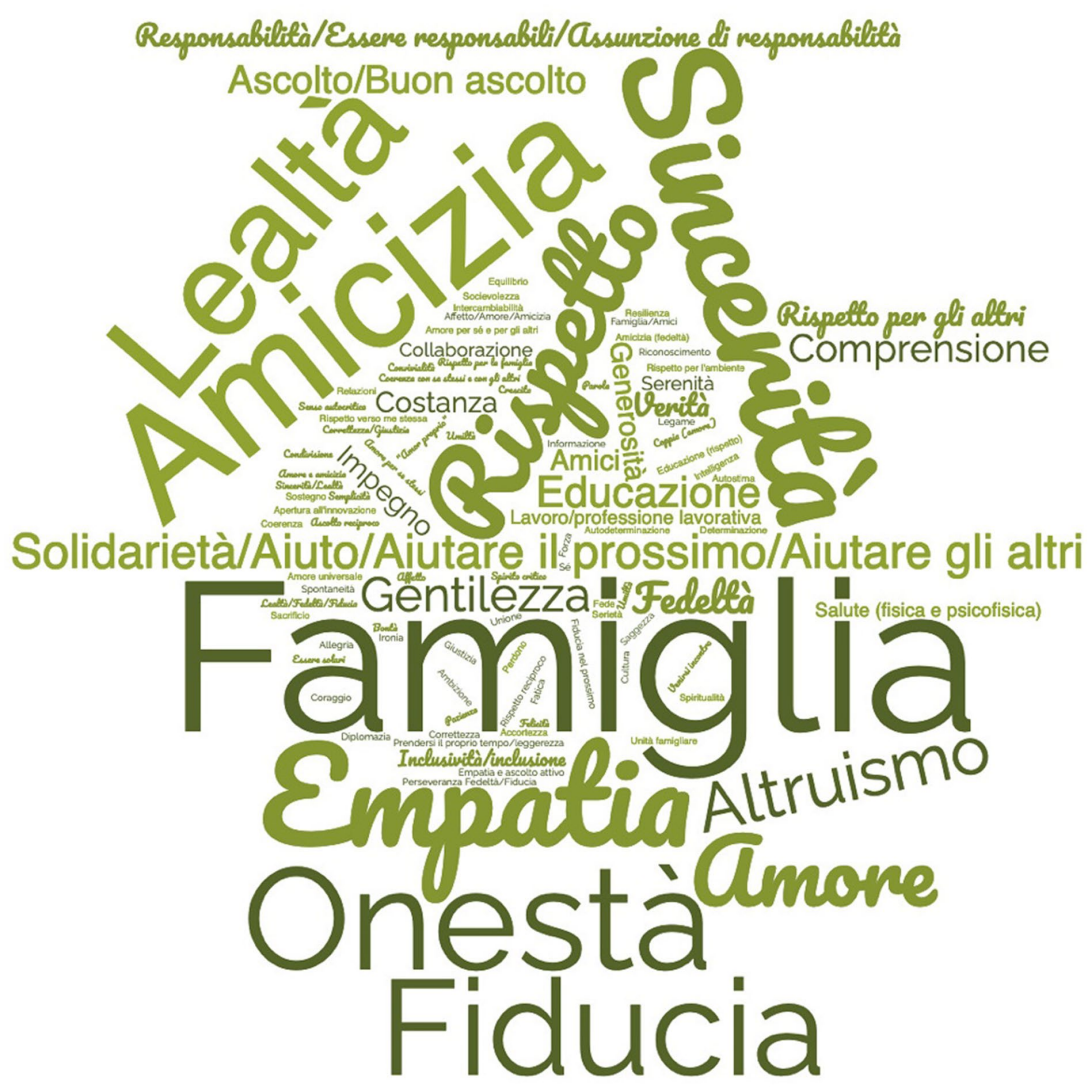
The value map.

Analyzing the entirety of preferences expressed by the adolescents, a total of 103 unique words emerged, accounting for aggregations and repetitions. Observing the WordCloud,^10^ we can note the centrality of “family” (27 preferences), which stands out as the most prominent value within the shared value framework of the participants. It is immediately followed by values such as “respect” (26), “friendship” (16), “honesty” (15), “trust” (14), “loyalty” (14), “sincerity” (14), and “empathy” (13; Figure 1).

In examining the values cited by each participant as “the most important” (Table 1), it becomes evident that “family” firmly maintains the top position, followed by “respect” and “honesty.” Friendship receives only two preferences, while the semantic domain surrounding “sincerity,” “solidarity,” “trust,” or “loyalty” appears to evoke the need to reconstruct the social “bond” that the pandemic seems to have compromised due to isolation and social distancing.

**Table 1:** Values considered most important.

Family	16
Respect	11
Honesty	6
Sincerity	4
Solidarity	3
Friendship	2
Trust	2
Education	2
Loyalty	2
Altruism	1
Love	1
Kindness	1
Consistency	1
Couple	1
Personal growth	1
Empathy	1
Balance	1
Health	1
Self	1
Seriousness	1
Truth	1

It is worth questioning whether the preference expressed for “family” as the most important value in the lives of adolescents represents a sincere connection to tradition and family bonds, or if it is rather an indicator of turning to the family as a source of economic security or a “safe haven,” not solely from an emotional standpoint. In the following paragraphs, we will explore whether the “traditional” values at the core of adolescents’ preferences are the same as those reflected in the media content they choose and engage with.

### From TikTok to BeReal: balancing entertainment and the need for authenticity among youth

As observed in the introduction, the media environment is now a frequent habitat for young people, serving as the space in which they construct identities, form relationships, and reinterpret social meanings. Within this increasingly expansive and fragmented media landscape, digital platforms, including social media and instant messaging apps, are currently the most frequented and appreciated. These platforms are perceived primarily as tools for engaging with novel realities that might not otherwise be accessible.

It is important to note that contemporary youth cultural consumption is situated within a transmedial context (Scolari, 2018), where content proliferates across multiple platforms, adapting and reshaping content across various formats and reconstituting cohesive symbolic universes.

During the pandemic, the use of these tools significantly increased, as reported by the participants. On some days, especially during inclement weather or when indoors due to cold, these platforms were used throughout the entire day. This data is consistent with other European studies on shifts in cultural consumption, which indicate an increase in consumption among young people: “increasing and diversifying cultural consumption during the pandemic was associated mostly with young age groups and was contingent on having spare time” (Feder et al., 2023, p. 51).

Foremost, young people refer to WhatsApp, which many consult immediately upon waking and use for communication with university mates and distant friends and relatives. WhatsApp has become an indispensable tool for young people, as expressed by one participant: “When I installed WhatsApp, I remember not wanting to, but I recall a statement from a friend: ‘We now only communicate through groups, so if you do not install it, you risk being left out’” [F (f)].

Other frequently used platforms include Instagram, TikTok, and Facebook, although the latter has largely been abandoned by young people in favor of newer media environments. TikTok, in particular, has become extremely popular among young people (Vizcaíno-Verdú and Tirocchi, 2021), even surpassing Instagram in their preferences, which is perceived as repetitive in its content. TikTok’s appeal lies in its entertainment value, personalization, and brevity. In terms of personalization, algorithmic mechanisms ensure that content is selected based on users’ preferences. However, TikTok is not just a platform for leisure but also a significant time-consuming activity for young people. One participant mentioned, “TikTok takes up a lot of my time because it keeps you scrolling all day. TikTok’s algorithm is perfect, which is why I spend two hours on it” [C (f)]. In fact, one interviewee confessed to uninstalling TikTok due to excessive usage.^11^

TikTok is also utilized as an educational tool, with users following nutritionists, gynecologists, and videos that provide quick insights into sexuality, filling gaps in knowledge not covered in formal education. The platform is also used to watch cooking videos and study methods demonstrated by other students.

For studying, young people turn to other social media platforms such as Instagram^12^ and YouTube, which remain valuable sources of information and tutorials on various topics, including politics and science. Some mention relatable content, referring to videos in which they recognize themselves in certain behaviors or situations depicted through memes, fostering identification. Memes, particularly “ironic memes,” defined as “meaningful digital multimodal text” (Ntouvlis and Geenen, 2023), are among the most appreciated cultural contents on Instagram. They serve to lighten a day filled with challenges or commitments. As one participant noted, “Memes are very fast; you do not have time to think about whether you like them. I used to make them, but I lost some of that enthusiasm because it takes time to create a meme that makes people laugh. You cannot produce something poorly made or low-quality” [G (m)].

Additionally, the youth discuss BeReal (2023), a smartphone app launched in 2020 in France by Alexis Barreyat and Kevin Perreau but gaining popularity among Generation Z, especially from 2022 (Maddox, 2023). As previously highlighted, this generation is particularly inclined to understand and experience the continuity between the online and offline realms. They are increasingly critical of online self-presentation, often perceived as disingenuous (Reinikainen et al., 2020; Maddox, 2023). BeReal has become a platform where users test their “authenticity” in ideological contrast with other social media platforms like Instagram or TikTok. Maddox observes, “For BeReal, authenticity is both discourse and value, emerging at a sociocultural moment in which questions about who people are online and how younger users engage with social media are ubiquitous” (Maddox, 2023, p. 2).

**Figure 2:**
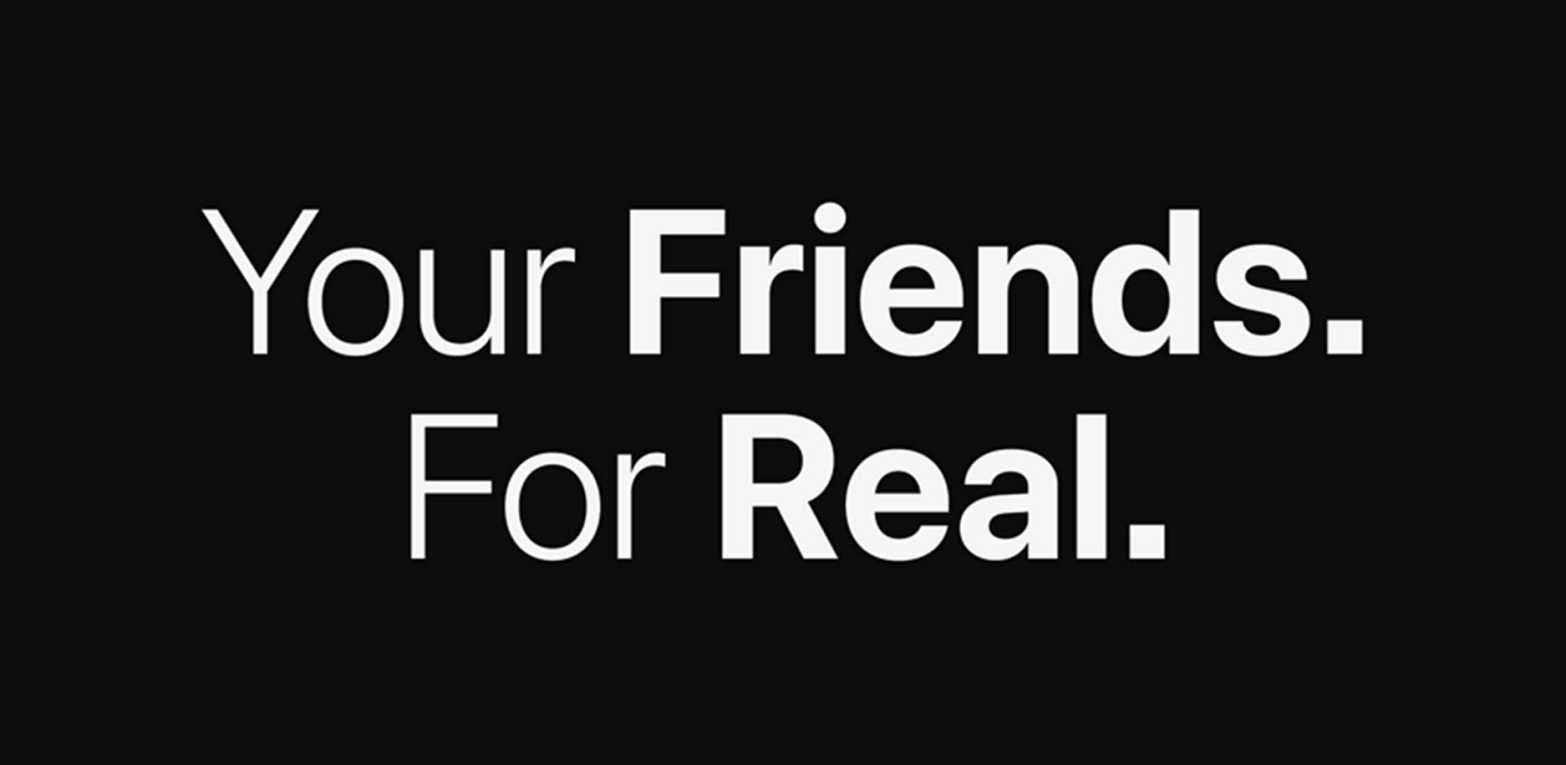
Slogan of the app BeReal. Source: https://bereal.com/.

BeReal focuses on the sharing of genuine, authentic moments from users’ daily lives. The app aims to promote transparency and authenticity, encouraging users to be themselves and share their lives genuinely and without filters. BeReal seeks to counter the trend of presenting an idealized and filtered version of users’ lives, with the aim of creating an inclusive and supportive community. Its main feature involves sending users a daily notification at a random time, inviting them to share a photo using both the front and rear cameras within 2 min. BeReal allows simultaneous selfie and rear-camera shots. As one participant described, “For example, I was at the supermarket buying “Estathé,” and I took a photo. Only those you add can see it” [G (f)]. BeReal photos can be shared, published, subject to challenges, comments, or emojis (Figure 2).

However, despite its popularity, BeReal is also criticized by some of its users. It is perceived as becoming “fake” because users are not obligated to take a photo at that moment. Many users wait to take photos during social events, which diminishes the authenticity of the platform. As one participant noted, “It became fake because you are not required to take a photo at that moment, so many wait to have an aperitif with friends, but in the end, the sense of reality is not there because maybe I’m at home studying and I do not take a photo. In any case, the medium creates a distance” [C (f)], compromising its authenticity claims to some extent.

### The universe of influencers and digital creators: between support and diversity

Within the realm of prominent figures and most-followed content, influencers have naturally found their place as reference points for young people, or at least for the majority of them, primarily due to their peer-to-peer style of communication, characteristic of Generation Z.

Beyond sporadic preferences for videos by Chiara Ferragni^13^ and Fedez, mainly followed due to interest in their daily routines and children, the influencers who garner preferences from young people are lesser-known creators who convey messages that are particularly resonant from a values perspective (see Table 2).

**Table 2:** Favorite media contents.

**Social media pages (Facebook, Instagram, TikTok, etc.), websites, and influencers**
Alessia Colonna (https://www.instagram.com/ale.colonna/)
Aprite il cervello (https://www.instagram.com/apriteilcervello/)
Arono Celeprin (https://www.instagram.com/aronohh/)
Associazione “Verba” (https://www.associazioneverba.org)
Bosnian History (https://www.instagram.com/bosniahistory/)
Charles Leclerc (https://www.instagram.com/charles_leclerc/?hl=it)
Commenti memorabili (https://www.instagram.com/commenti_memorabili/?hl=it)
Cucina con Ruben (https://www.instagram.com/cucinaconruben/)
Diariodiunaeducatrice (https://www.instagram.com/diariodiuneducatrice/?hl=it)
Emilife (https://www.instagram.com/emanueleferrari/?hl=it)
Emily Pallini (https://www.instagram.com/emilypallini/)
Eleonora Petrella (https://www.instagram.com/elepetrella/?hl=it)
Factanza (https://www.instagram.com/factanza/)
Fanpage (https://www.fanpage.it)
Fedez (https://www.instagram.com/fedez/?hl=it)
Flavia Carlini (https://www.instagram.com/flavia.carlini/)
Francesco Cicconetti (https://www.instagram.com/mehths/?hl=it)
Giallozafferano (https://www.giallozafferano.it)
Gianluca Gotto (https://www.mangiaviviviaggia.com)
Giulia Azzolini (https://www.instagram.com/giulia.azzolini/?hl=it)
GiuliaLaMarca (https://www.instagram.com/giulialamarca/?hl=it)
Giulia Zollino (https://www.instagram.com/giuliazollino/)
Giovanni Arena (https://www.instagram.com/giovanniarena_/)
Iovivolasclerosimultipla (https://www.instagram.com/iovivolasclerosimultipla/?hl=it)
Italia Team (https://www.instagram.com/italiateam/)
La storia di Cesare (https://www.instagram.com/la_storia_di_cesare/)
Luca Argentero (https://www.instagram.com/lucaargentero/)
maybeSimona (https://www.instagram.com/maybesimona/?hl=it)
Mattia Stanga (https://www.instagram.com/mattia_stanga/?hl=it)
Nicolas Maupas (https://www.instagram.com/nicolas_maupas/?hl=it)
Notizie.it (https://www.notizie.it)
Raissa e Momo (https://www.instagram.com/raissarussi/; https://www.instagram.com/mibayed/ targatoCn (targatoCuneo)
Vaneblandy (https://www.instagram.com/vaneblandy/)
Utravel (https://utravel.it)
Webboh (https://www.webboh.it)
Will (https://willmedia.it)
Wordly (https://www.beworldy.com)
**Tv series**
Brooklyn 99 (Fox, NBC, 2013–2021)
Chicago fire (NBC, 2012-in production)
Dahmer—Mostro: la storia di Jeffrey Dahmer (Netflix, 2022)
Doc—Nelle tue mani (Rai1, 2020-in production)
Dr. House (Fox, 2004–2012)
Élite (Netflix, 2018-in production)
Friends (NBC, 1994–2004)
Full House (Netflix, 2016–2020)
Grey’s Anatomy (ABC, 2005-in production)
How I met your mother (CBS, 2005–2014)
Maid (Netflix, 2021)
Manifest (NBC, Netflix. 2018–2023)
Mare fuori (Netflix, Raidue, 2020-in production)
Mercoledì (Netflix, 2022-in production)
Narcos (Netflix, 2015–2017)
Suits (USA Network, 2016–2019)
The Crown (Netflix, 2016-in production)
The Good Doctor (ABC, 2017-in production)
The Last Dance (ESPN, Netflix, 2020)
The Vampire Diaries (The CW, 2009–2017)
This is Us (NBC, 2016–2022)
Una mamma per amica (2000–2007, WB)
**Film**
A beautiful mind (2001)
Fight Club (1999)
Forrest Gump (1994)
Il diritto di contare (2016)
Il signore degli anelli (2001–2003)
Dom Durakov—La casa dei matti (2002)
Patch Adams (1998)
Spiderman into the spiderverse (2018)
**Books and writers**
Nicholas Sparks
**Music (singers, bands, etc.)**
Eugenio in via di Gioia
J-Z
J-AX
Pink Floyd
**Tv programs**
Boez—Andiamo via
Chi l’ha visto
Home & Garden
L’eredità
Maratona Mentana
National Geographic
Piazza pulita
Un giorno in pretura
90 giorni per innamorarsi
**Anime/Manga (comics, cartoons, TV series)**
Naruto
My Hero Academia
One Peace
**Youtube channels**
Canale di Venti (https://www.youtube.com/c/canalediventi)
Elisa True Crime (https://www.youtube.com/c/elisatruecrime)
Geopop (https://www.geopop.it)
Mondo dei bambini (https://www.youtube.com/channel/UCvebr2jW6cPTlKeMDSJ_84g)
Inntale (https://www.inntale.com)
L’aiuto qui e ora (https://www.eleonoraferraro.it/laiuto-qui-e-ora/)
L’occhio Creepy di Youtube (https://www.youtube.com/@OcchioCreepy)
Lo strano canale—True Crime (https://www.youtube.com/c/LOSTRANOCANALE)
(https://www.youtube.com/results?search_query=lo+strano+canale)
Nova Lectio (https://www.youtube.com/@NovaLectio)
Studywithme (https://www.youtube.com/playlist?list=PLbpi6ZahtOH5fWoEOOV_hGarI4FxKHULy)
Ted Talk (https://www.youtube.com/channel/UCAuUUnT6oDeKwE6v1NGQxug)

Young people’s preferences can be grouped into seven themes covered by influencers, creators, and web pages (see Table 3).

**Table 3:** Values and issues emerged from influencers and digital creators (mentioned in the focus groups).

Sexuality and LGBTQ+ Issues
Disabilities
Authenticity, Genuineness, and Sustainability
Digital Nomadism, Travel, Nature
True and Reliable Information
Comedy and Entertainment
Personal Growth, Psychology, Education

#### Sexuality and LGBTQ+ issues

This content category aligns with sensitivities regarding gender-related issues, LGBTQ+ issues, gender-based violence (with the rise of femicide), or racism. Pages that pay attention to sources and use simple language are appreciated. Examples in this content category include Giulia Azzolini, a sexologist who delves into various types of relationships beyond the “traditional” ones, as one participant noted: “We never received relational and emotional education. She opens up new perspectives on life and relationships” [L (f)]. Other mentioned figures include Francesco Cicconetti, a transgender individual, and Arono Celeprin, who identifies as non-binary. There is also a couple, Raissa and Momo, an Italian and Moroccan pair, who have sought to challenge prejudices and promote “kind communication” on social media, using humor to address racism.

#### Disabilities

An example in this category is the profile of Giulia La Marca, a blogger and digital creator passionate about travel, who became disabled following a moped accident. She showcases how seemingly insurmountable obstacles can be overcome. In the same thematic area, young people mention the social page “La storia di Cesare,” managed by the mother of a child who became blind due to a tumor.

#### Authenticity, genuineness, and sustainability

The qualities that young people wish to find in media, especially on social media, revolve primarily around spontaneity and authenticity: “Nobody has a perfect life. [I would like to] see a real life that does not come from social media or reality shows” [S (f)]. They seek a life “without filters,” much like what is portrayed by influencers like Emily Pallini, who, despite having acne, chooses to show herself without filters and accepts herself as she is. An example of an influencer discussing sustainability is the microcelebrity Simona (*maybeSimona*).

#### Digital nomadism, travel, nature

Young people are fascinated by the profiles of travel bloggers who work remotely, often referred to as “digital nomads” (Cook, 2023). These individuals often have blogs or e-commerce businesses, their own online companies, and earn income by promoting their content on social media. This allows them to work from anywhere in the world. These influencers are appreciated for their ability to “draw inspiration to pursue their dreams… Love their work because it’s based on their passion” [D (f)]. An example cited in focus groups is Gianluca Gotto, an Italian who abandoned everything and, along with his girlfriend, travels the world and has written two books related to travel. “I felt like just packing up and leaving everything” [S (f)]. Another name mentioned is Giovanni Arena, a TikToker and travel creator. The love for travel is often accompanied by a passion for nature and landscapes.

### True and reliable information

Information on social media is highly appreciated for being perceived as more direct, faster, and less biased compared to traditional sources. For instance, “Fanpage” news, which focuses on topics relevant to young people rather than politics, is cited, as is the Instagram page “Aprite il cervello,” addressing issues such as immigrant rights and LGBTQ+ rights. Flavia Carlini is also mentioned as a good example of informative content, and in a similar style, young people mention the culture page “Will” and the Instagram profile “Factanza.”

#### Comedy and entertainment

An example of this diverse category is Mattia Stanga’s videos, in which he humorously assumes the role of his mother. Also included in this preference group are the aforementioned ironic memes.

#### Personal growth, psychology, education

Participants in the study appreciate content related to personal growth and professional figures who can serve as sources of inspiration for their careers, such as educators, pedagogists, and experts. Particularly of interest to girls are profiles of mothers who post photos of their children.

In analyzing the preferences expressed by the students, numerous differences are observed when compared with their parents and siblings.

Parents, in most cases, still primarily turn to mainstream television, except for rare occasions when they spend time on apps like TikTok. There are mentions of “anti-technology” or technologically challenged fathers who can barely use WhatsApp. On the other hand, there are 80-year-old grandparents who want to venture into the world of technology, as recounted by a young interviewee: “My 80-year-old grandfather wants to learn how to use a computer, instead” [C (f)].

A phenomenon is also noted that reflects a significant difference between the youth and their younger siblings, a style of social media management that is much more private and operates on two levels. “All of my sister’s friends (who are 17 years old) have two profiles. In one, they hardly post anything, while in the other, they share stories or moments they spend together” [A (f)]. A similar observation is made: “At 17, my younger sister’s friends have two Instagram profiles, one ‘private’ (where not everyone is accepted) and one open to all” [M (f)]. The way they use social media is notably different because younger individuals tend to post fewer photos and prioritize the so-called “highlight stories.” In their “private” profiles, where they exchange photos and posts, they have only 20, 30, or a maximum of 40 friends.

As one participant in the focus group observes, “If, once upon a time, young people were escaping from Facebook, now adults have also arrived on Instagram […]. Now everyone is there: teachers, parents. There is a need to escape and find another reality that escapes the control of adults” [G (m)].

### TV series: new narratives for identification

TV series represent a highly favored form of media content among young people.^14^ They appreciate the ability to consume content in a highly personal manner, at any time, and according to their preferences on specific on-demand platforms. Netflix, in particular, stands out among these platforms, as it is making an effort to emphasize diversity (Asmar et al., 2023). Perhaps for this reason, it holds an appeal for Generation Z, offering themes and issues that resonate with the younger generation.

In the words of G [(m)], a preference for this media language emerges: “I used to hate TV series because I was used to watching movies with a clear beginning and end… It bothered me that they did not conclude. Now it’s become almost an addiction….” Series are often watched together, for example, with the housemates.

Furthermore, according to research participants, the slower pace of TV series allows for a deeper exploration of the characters’ psychology.

There are various types of TV series favored by young people. These range from fun or relaxing series, watched as a means to take a break from studying, often centered around humor, such as “Brooklyn 99” (Fox, NBC, 2013–2021), to series like “Suits” (USA Network, 2011–2019), which tells the story of a prestigious law firm in New York. There is also a resurgence of “classic” series like “Friends” (NBC, 1994–2004), which is chosen for the values it conveys [G (f)]: friendship, humor, family, and children. Also in the realm of family themes is “Maid” (Netflix, 2021), a series that features a single mother who must “make it on her own” to survive. Mentioned in the context of family are “Una mamma per amica” (2000–2007, WB) and “This is Us” (NBC, 2016–2022), the latter being highly appreciated for addressing themes such as self-acceptance, adoption, and loss. “The Crown” (Netflix, 2016-in production) is appreciated for its perceived ability to make history more engaging.

Another highly appreciated genre is thriller: series that revolve around murders or even docufiction, characterized by a blend of fiction and reality.

The series “Mercoledì” (Netflix, 2022-in production) is chosen for its ability to convey values such as the determination of the girl who is not influenced by others’ opinions. In “Élite” (Netflix, 2018-in production), characters are portrayed authentically, showcasing all their flaws, such as acne or cellulite.

Another genre that highlights the importance and strength of the group is that of anime and manga, including “Naruto,” “My Hero Academia,” and “One Piece.”

Finally, when looking at traditional media, it becomes evident that films still hold their ground as a format appreciated primarily for their self-contained nature. The favorite films are often classics, not contemporary ones for the young people, but they continue to convey very specific values (a prime example being “Forrest Gump”).

## Conclusions and perspectives: desire for authenticity and emerging values.

The study presented in this article has highlighted the challenge of conducting research on the relationship between values and media in a society that is increasingly unstable and complex, as described by our Generation Z interviewees as a “society without values” or one witnessing a true “disintegration of values.”

The long tradition of quantitative research on the measurement of values (Roccato, 2008), particularly among young generations, has undoubtedly provided an essential foundation from which to develop further insights. In this case, we chose to adopt a qualitative approach based on the use of focus groups, during which we sought to elicit the opinions and attitudes of young people on the investigated topic.

It is important to emphasize that the research is not generalizable but rather focused on a specific context, that of Education Sciences students at the University of Turin, with a predominance of female participants. This aspect constitutes a limitation of the research that does not allow the results to be extended to the entire Italian population. In this sense, the survey takes on a specifically exploratory character that can nevertheless form the basis for future in-depth studies.

However, a strength of the study lies in its exploration of the relationship between young people’s values and the values conveyed by media content, a topic that has been underexplored in scientific literature, particularly in sociology.

Concluding the analysis of values declared by the young people and those emerging from the most cited cultural contents, we can draw some preliminary conclusions that may serve as a starting point for further research.

The set of values “declared” by young people and deemed most important highlights a persistence of traditional ethical references, starting with the unequivocal centrality of the family. However, the list of values that indirectly emerge through the media content they choose and highlight in their discussions suggests both similarities and differences. Indeed, young people choose values such as acceptance of disability, environmental concern and sustainability, personal growth, and enjoyment.

One aspect particularly appreciated by young people lies in the pursuit of authenticity and truth in media content, especially reflected in the preference expressed by some for the app that is expected to embody these qualities, BeReal, the application that promises to capture and immortalize genuine and authentic moments from our day. Along the same line of thinking, young people prefer movies that tell “true stories” or documentaries based on images and testimonies. Things they dislike are those that embody fiction and represent the opposite of reality, such as reality television.

An evident signal of attention to “reality” and “authenticity” concerns the importance of information based on “fact-checking” in an era dominated by fake news. However, young people do not seek this attention from traditional sources (parents, teachers, broadcast media) but from new peer-to-peer mediators and influencers who express themselves through YouTube channels or Instagram pages, where they offer information (at least seemingly) free from biases.

The interaction between the values of young people and the values conveyed by the media is indeed a complex and ever-evolving theme. We know that the media can inevitably influence the values of young people in various ways, and, even more so, social media, starting with their widespread use, have provided young people with a space for visibility and self-representation that still allows them to navigate between a dimension of authenticity (Schwarz and Williams, 2021) and one of “false representation,” deciding what to post or share, when to do it, and how to do it (Boyd, 2014; Marwick and Boyd, 2014) and skillfully using both “authentic” and “fake” profiles (Darr and Doss, 2022).

The research has shown, perhaps unexpectedly, that instead of focusing on self-promotion or polished and flawless self-representation on social media, young people choose or claim to appreciate authenticity, genuineness, at the expense of false representations. In this regard, critical media literacy can help develop the ability to evaluate media content based on one’s personal values or to use media for self-representation or content creation in a conscious and responsible manner (Tirocchi, 2013; Buckingham, 2020).

## Data availability statement

The raw data supporting the conclusions of this article will be made available by the author, without undue reservation.

## Ethics statement

Ethical approval was not required for the studies involving humans because any data used in the study was collected and analyzed in a way that ensured the complete anonymity and privacy of individuals. No personally identifiable information was used or disclosed. The studies were conducted in accordance with the local legislation and institutional requirements. The participants provided their written informed consent to participate in this study.

## Author contributions

ST: Writing – original draft, Writing – review & editing.

## Footnotes

^1^Refer to, in particular, the latest published reports: Buzzi et al. (1997,^2002^,2007).

^2^The Giuseppe Toniolo Institute, the founding institution of the Catholic University, has been producing the Youth Report on an annual basis since 2012, offering a snapshot of the youth landscape in Italy. This report is the result of a collaboration with the Statistics Laboratory of the Catholic University, with the support of the Cariplo Foundation and in partnership with IPSOS S.r.l. serving as the executive partner. The findings of this survey are annually published by Il Mulino (Bologna). In 2021, the third three-year cycle of the longitudinal study representing young Italians was initiated, with a projected sample size of 7,000 interviews. The Youth Report and its associated Observatory provide data, analysis, and insights into the youth demographic, with the aim of focusing public attention on these issues and guiding policies, services, and initiatives.

^3^In this scale, 0 represents “not at all more at risk,” while 5 represents “extremely more at risk.”

^4^The National Recovery and Resilience Plan (PNRR), introduced in Italy in response to the global COVID-19 emergency, is part of the European Next Generation EU project. This program entails the allocation of resources amounting to 50 billion euros for the purpose of stimulating growth, investments, and reforms. Of this total, more than half, 390 billion euros, consists of grants, marking a significant departure from previous decades.

^5^See also, regarding theories on the social effects of the media: Belluati and Tirocchi (2023).

^6^For a reflection on recent definitions of generation, see also: Tirino (2022).

^7^In regard to the definition of the concept of generation, a fundamental contribution comes from Mannheim (1928). For further insights on this topic, refer to Merico (2012,2019).

^8^The concept of the “prosumer” developed by Toffler (1981) had anticipated the collaborative content creation processes that later characterized social media and the web 2.0. Bruns observes how Toffler’s prosumer model remains deeply rooted in the world and era of mass media. According to Bruns, projects like Wikipedia and other communities characterized by creative and participatory content production demonstrate that the roles of the consumer and user are inextricably intertwined with those of the producer and creator. This new hybrid role can therefore be better described, according to the author, by the term “produser” (Bruns, 2008).

^9^Survey conducted from 14 April to 4 May 2022. The respondents involved in the study were between the ages of 13 and 17.

^10^The WordCloud was created using https://www.wordclouds.com/. It was chosen to keep the terms indicated by the adolescents in Italian.

^11^These considerations prompt reflection on the so-called detox culture. In one instance, a young man [G (m)] declares not to possess any social media, a choice he made during the pandemic period: “I had Instagram, Facebook; I spent a lot of time during the pandemic scrolling… and at a certain point, I said to myself, ‘Man, I’m here scrolling, could I be doing something else?’ I thought: since I have the power over these apps, I’ll uninstall them. The problem is that today, I still spend my days on YouTube, where I watch videos or there are shorts, mini videos (30 s), because the shorter they are, the more inclined you are to watch many; they appear immediately, right below the YouTube page.”

^12^See also Taddeo (2023), on Instagram as an informal learning environment.

^13^First and foremost, we recall the book written by Ferragni (2013) herself, titled “The Blonde Salad: Style Tips from the Most Followed Fashion Blogger on the Web.”

^14^On the topic of TV series, with a specific focus on the use of clothing, see: Mascio (2023).
